# Assessment of Occupational Health and Job Satisfaction in Workers with Intellectual Disability: A Job Demands–Resources Perspective

**DOI:** 10.3390/ijerph18042072

**Published:** 2021-02-20

**Authors:** Noelia Flores, Carmen Moret-Tatay, Belén Gutiérrez-Bermejo, Andrea Vázquez, Cristina Jenaro

**Affiliations:** 1 Institute on Community Integration (INICO), Department of Personality, Assessment and Psychological Treatments, Faculty of Psychology, University of Salamanca, 37005 Salamanca, Spain; crisje@usal.es; 2 Faculty of Psychology, Catholic University of Valencia-San Vicente Mártir (UCV), 46100 Burjassot-Valencia, Spain; mariacarmen.moret@ucv.es; 3 Department of Neuroscience, Mental Health and Sense Organs (NESMOS), Sapienza University of Rome, 00185 Rome, Italy; 4 Faculty of Psychology, National University of Distance Education (UNED), 28040 Madrid, Spain; mbgutierrez@psi.uned.es; 5 Faculty of Psychology, International University of Valencia (VIU), 46002 Valencia, Spain; andrea.vazquez.m@campusviu.es

**Keywords:** Job Demands–Resources theory, work-related factors, exhaustion, work engagement, job satisfaction, intellectual disabilities, assessment

## Abstract

In the contexts where people with intellectual disability work, there are factors that determine their job satisfaction. The objective of this study was to test the adequacy of the central assumptions of the Job Demands–Resources (JD-R) theory in workers with intellectual disability employed in different work alternatives. Data from 362 workers in sheltered workshops and 192 workers in supported employment were utilized. The model was contrasted using a structural equation model and a multi-group analysis. The results supported the suitability of the model and confirmed that job demands and job resources evoke two relatively independent processes such as health impairment and motivational process. The multi-group analysis confirmed the invariance of the model between the two work alternatives. Thus, the JD-R model offers a useful framework to explain the job satisfaction of workers with intellectual disability. Implications for the improvement of personal and job results are discussed.

## 1. Introduction

Employment is one of the main activities of industrialized societies and an essential factor in ensuring equal opportunities through the participation of citizens in economic, social, and cultural life [1,2]. Work organizes people’s lives and provides income. Numerous studies have also shown that work promotes well-being and provides a means for individual satisfaction and achievement [3,4,5,6]. Thus, it can be said that for most, work represents a fundamental dimension of human existence.

People with intellectual disability (ID) are not an exception. Intellectual disability includes different conditions classifiable according to the International Classification of Diseases ICD-11 [7] as disorders of intellectual development (6A00). These disorders are a group of etiologically diverse conditions originating during the developmental period characterized by significantly below average intellectual functioning and adaptive behavior. A similar definition is included in the DSM-5 manual [8] where the condition is called intellectual disability. According to the International Classification of Functioning, Disability and Health (ICF), this condition is related to limitations in general mental functions, which are required to understand and constructively integrate the various mental functions including all cognitive functions and their development over the life span [9]. Finally, The American Association on Intellectual and Developmental Disabilities (AAIDD) [10] defines it as a disability characterized by significant limitations in both intellectual functioning and in adaptive behavior that covers many everyday social and practical skills. This disability originates before the age of 18. All these definitions share common elements to consider that this condition implies limitations that require support to guarantee the same rights as for the general population.

In relation to employment, the Convention on the Rights of Persons with Disabilities [11] recognizes “the right of persons with disabilities to work, on an equal basis with others; this includes the right to the opportunity to gain a living by work freely chosen or accepted in a labor market and work environment that is open, inclusive, and accessible to persons with disabilities” (p. 19). The 2030 Sustainable Development Agenda is based on the commitment to leave no one behind. To achieve this goal, the full inclusion and effective participation of people with disabilities in society and development are necessary [12]. Thus, the objective 8.5 of Sustainable Development aims “by 2030 to achieve full and productive employment and decent work for all women and men, including young people and people with disabilities, as well as equal remuneration for work of equal value” [13].

The European Parliament, on its resolution on the European Disability Strategy post-2020 [14] states that employers must be supported and encouraged to ensure that persons with disabilities are empowered. It calls on the commission to ensure that the post-2020 strategy will especially promote guaranteed access to employment. It also calls on the commission to recognize, promote, and protect inclusive enterprises to create permanent employment for people with disabilities in the labor market, stresses the potential of social economy enterprises and organizations to facilitate labor market inclusion for persons with disabilities, and calls on the commission to provide targeted support from the European Social Fund for the social economy. However, the employment rates of this group are still significantly lower than those of the non-disabled population or those with other types of disabilities (e.g., physical or sensory). Consequently, people with ID are underrepresented in the labor force [15,16,17]. Nevertheless, an increasing number of adults with ID are entering employment and engaging in productive activity [18,19], either within normalized, community-integrated enterprises or in sheltered employment [20,21].

For more than two decades, numerous studies have shown that people with ID can successfully perform work and contribute to the community [16,22,23,24,25,26]. In addition, these individuals also report their satisfaction, and most indicate that they are highly motivated to work [27,28,29]. In turn, work has been found to improve self-esteem, self-determination, social inclusion, social and professional networks, income, health, well-being, and quality of life [22,25,26,30,31,32]. All this underlines the importance of obtaining and maintaining employment for this group. Consequently, for decades, the literature has been more focused on assessing the benefits of having a job [33,34,35,36] rather than on analyzing the characteristics of the working environment, which contribute to personal and organizational outcomes [37]. In most cases, studies did not ask the person with ID about his/her own work experience. Instead, they gathered information from third parties (supervisors, job coaches, or people from the family environment). This has been considered one of the main weaknesses of research in this field, as the opinions of these workers were overlooked in most studies [15].

Nowadays, research on these topics is still scarce, and there is a need to increase knowledge regarding the factors in the work environment that enhance or hinder occupational health and satisfaction, from the perspective of the worker with an intellectual disability. Over the last decade, there have been numerous attempts to fill this research gap. In fact, an interesting body of work has emerged whose aim has been to analyze the characteristics and factors in the work environment related to the well-being and satisfaction experienced by workers with ID [38,39,40,41,42,43,44]. Job satisfaction has been one of the most analyzed constructs within organizational psychology [45]. In the field of intellectual disability, it is also a relevant topic where a recent systematic review concluded that the factors involved in job satisfaction of workers with ID are the same as those found in the non-disabled population [46]. However, studies in this group are scarce, and further research is needed [20].

The most widely used definition of job satisfaction was proposed by Locke [47], who defines it as “a pleasurable or positive emotional state resulting from the appraisal of one’s job or job experience” (p. 1300). This definition emphasizes the affective dimension of the construct while incorporating cognitive and attitudinal elements. Therefore, many interrelated agents impact a worker’s experienced job satisfaction. Job satisfaction is associated with personal (e.g., expectations, dispositional factors, sociodemographic), situational (e.g., job characteristics, social supports, physical and psychological demands), and socio-cognitive (e.g., the appraisal of these job factors) characteristics.

Although earlier research on this matter in the field of intellectual disability emerged at the end of the 1980s [48], having a job, especially in integrated contexts, was considered reason enough to experience job satisfaction. In fact, a large body of research concluded that people with ID who worked in integrated employment experienced greater job satisfaction than their peers who worked in more segregated environments, or unemployed people from the general population [26,34,49,50].

Satisfaction also seems to depend on personal factors, as well as on characteristics and conditions in which the work is done but, above all, on the appraisal of the workers themselves of these factors, in particular the nature of the tasks, working conditions, physical and psychological demands, social relations, and perceived support from colleagues and supervisors. Likewise, features such as the perception of autonomy, self-determination, decision-making, independence, and motivation have been found to be related to job satisfaction in the scarce research carried out in this field [19,39,40,51,52,53]. Those features, when perceived negatively, can become stress-inducing risk factors and jeopardize worker health and organizational outcomes. Very few studies have analyzed the influence of job characteristics on the stress experienced by workers and its impact on work well-being. In these cases, it has been shown that workers with ID who perceive job demands more intensely also experience greater job stress, which negatively impacts their productivity and quality of work life [41,54,55].

Therefore, in order to determine the type of support that people with ID need in their workplace, it is necessary to deepen the study of job satisfaction and associated factors. It is also advisable to adopt an integrative and multidimensional framework that will allow determining the nature of the associations between its variables. The Job Demands–Resources Theory (JD-R), proposed by Demerouti et al. [56] provides an appropriate framework. Its application to the field of intellectual disabilities is still rather limited [20,46].

### Job Demands–Resources (JD-R) Theory

The JD-R theory is an extension of the Job Demand–Resources model [56,57] which, in turn, draws on the theories of job design and occupational stress [58]. It is a theoretical model that helps understand, explain, and predict aspects of employee well-being and job performance, including job satisfaction [58,59]. One characteristic of JD-R theory is its flexibility [60]. This allows it to be applied in any occupational environment, to any professional group and detect the consequences of those specific environments on occupational health and performance, allowing for interventions to improve the well-being of employees. Therefore, its application in the field of intellectual disability is relevant for the advancement of research in this field of study.

The theory is based on a series of specific propositions [61]. The first assumption establishes that work environments and characteristics can be divided into two categories: job demands and job resources. Job demands refer to those “physical, psychological, organizational or social aspects of the job that require sustained physical or mental effort and are therefore associated with certain physiological and psychological costs” [56] (p. 501). Job demands can be an obstacle when they require a high effort from the worker [58]. Job resources refer to “physical, psychological, organizational or social aspects of work that can: (a) reduce job demands at the associated physiological and psychological costs, (b) be functional in achieving work goals, or (c) stimulate personal growth, learning and development” [57] (p. 312), [56] (p. 501). Job resources are not only necessary to meet demands but also have their own effect [58]. Social support from coworkers or supervisors, obtaining feedback on performance, and having autonomy are examples of resources. Numerous investigations with ID workers have identified job demands and resources associated with their well-being and job satisfaction [19,39,40,44,51,52,53,54,55].

A second proposition of the JD-R theory is that job demands and job resources develop two relatively independent processes, namely: health impairment and motivational process. The first process occurs in situations where high job demands and low job resources leads to exhaustion, which is the core dimension of burnout [62]. This is considered the most important predictor of the negative impact of stress on worker’s health [63]. Thus, prolonged exposure to occupational demands produces a negative effect on health and organizational outcomes through burnout. Thus, in accordance with this proposition, we predict the following:

**Hypothesis 1** **(H1).** *Job demands will be negatively related to job satisfaction through their impact on exhaustion (i.e., core dimension of burnout).*

On the other hand, the motivational process relates to job resources. For example, adequate job resources stimulate worker motivation and leads to engagement which, in turn, generates positive work outcomes that impact on the individual and the organization (e.g., through increased work performance and commitment). Job resources are the most important predictors of engagement [60,62,64]. This construct is defined as a “positive, work-related state of mind characterized by vigor, dedication and absorption” [65] (p. 4), with vigor and dedication being the core dimensions of engagement [60,62,66]. Therefore, work environments that promote resources stimulate the intrinsic motivation of workers and, in turn, increase, their involvement and effort toward tasks, thus achieving greater well-being and better organizational results. Thus, we predict the following:

**Hypothesis 2** **(H2).** *Job resources will be positively related to job satisfaction through their impact on work engagement (with vigor and dedication as the main motivational process).*

These two processes have been identified in empirical and meta-analytical studies [62,66,67,68,69,70]. Despite evidence that workers with intellectual disability may experience burnout and engagement in their workplaces [54], no research has replicated this dual pathway proposed in the JD-R model. The recent work carried out by Ybema et al. [55] is the only exception, although they use a non-representative sample of workers with ID, which makes it difficult to generalize.

JD-R theory also postulates that job demands and job resources interact in predicting well-being. Here, resources buffer the impact of demands on occupational health and well-being; that is, employees with more resources will better cope with workplace demands and will experience less discomfort and exhaustion. Some studies on workers without disabilities have shown that having work resources can lessen the impact of demands on exhaustion [71,72]. However, support for this proposition has not always been found [59,67]. The few studies conducted with workers with disabilities also provided contradictory results [54,55], so further research into these relationships is required. Accordingly, we predict the following:

**Hypothesis 3** **(H3).** *Job resources will reduce exhaustion.*

The robustness of this model has been demonstrated in numerous studies and with different professional groups [59,66,67,69]. A recent meta-analysis of longitudinal studies suggest that the model provides an excellent theoretical basis for assessing employee well-being in any professional field [70]. However, the JD-R model has been sparsely used. In the study by Flores at al. [40], it was used to analyze quality of work life in workers with intellectual disability in sheltered workshops and supported employment. Likewise, Akkerman et al. [39] used it to understand the influence of job characteristics and personality traits on the job satisfaction experienced by workers with ID. Finally, Ybema et al. [55] utilized it as a framework to explain the well-being and productivity of workers with diverse disabilities in sheltered employment. All these studies assessed the limited variables of the model [20,40] or utilized insufficient and unrepresentative samples [55]. Therefore, it is necessary to increase research and provide evidence on the applicability of the JD-R model to workers with intellectual disability, using larger samples belonging to the two most common employment modalities in the hiring of this group (sheltered workshop vs. supported employment). This would make the theory more robust and will allow the design of specific proposals for intervention to improve the occupational health of this group.

This work aims to use the Job Demands–Resources theory (JD-R) [56,58,61] with a large group of workers with ID belonging to the two most used employment modalities in the hiring of this group (sheltered vs. integrated). More specifically, this study aims to investigate the fit of the two-way process established in the JD-R theory to explain the job satisfaction of these workers. It also aims to explore its appropriateness and validity in the prediction of well-being and job satisfaction, using the procedure of structural equations. An additional aim is to demonstrate the appropriateness of some of its fundamental postulates [58,61]. Therefore, in accordance with previous predictions derived from JD-R theory, we also predict the following:

**Hypothesis 4** **(H4).** *The health impairment and the motivational process stated in JD-R theory will function similarly in both employment modalities under study.*

## 2. Materials and Methods

### 2.1. Participants and Procedure

The participants in this study were recruited through state and local supported employment agencies as well as from sheltered workshops. The inclusion criteria were as follows: (1) a diagnosis of intellectual disability (mild to moderate) [8] as confirmed by the different centers or agencies; (2) sufficient communication skills, as judged by agency personnel, to understand and respond to the questionnaires; (3) being between 18 and 65 years old; (4) at least 3 months of continuous employment in the present position; and (4) volunteered participation in the study, after informed and written consent.

A convenience sample of 554 workers with intellectual disability from eleven sheltered workshops and eight supported employment initiatives from Spain took part in this study. All the centers and companies in which the participants with ID developed their work activity were located in urban centers belonging to the Autonomous Communities of Catalonia (37.9%), Madrid (22.7%), Cantabria (20.8%), and Andalusia (18.6%). Participants were fully informed about the aims of the study during previous meetings to help clarify any questions.

Of the total, 58.6% were men and 41.4% were women. The percentages for age range in years were as follows: 4% for 16–21, 33.8% for 22 to 30, 36.9% for 31 to 40, 19% for 41 to 50, 6% for 51 to 60, and 0.4% for over 60 years old. With regard to marital status, 92.6% were single, 6.1% were married or living with their partner, 0.5% were widowed, and 0.7% were divorced. A total of 10.1% of the participants had to manage work with other responsibilities. With regard to the type of employment, a total of 362 (65.3%) worked in sheltered workshops, while 192 (34.7%) belonged to different supported employment initiatives in the community. With regard to work conditions, a total of 74.6% had a permanent contract, and 25.4% had a temporary contract. Concerning length of employment, 29.6% of the workers were employed for more than 11 years, followed by those working less than 1 year (17.6%) and by those working for 3 to 5 years (16.7%). Regarding job positions, they included assembling, cleaning, gardening, packing, carpentry, clothing manufacturing, shop assistance, and office work.

A cross-sectional study design was used to assess job demands, job resources, exhaustion, work engagement, and job satisfaction. Data were collected from March to November of 2016. Telephone and email were used to establish contact with the different organizations, followed by visits to the centers to conduct the assessment via individual interviews. Participants were visited at their workplace or at the employer’s offices for supported employment by two of the authors (N.F. and C.J.). The average length of the interviews was 60 to 75 min. Anonymity and confidentiality were guaranteed. In order to ensure anonymity and protect the personal data of the respondents, sociodemographic data were recorded separately from the response data.

### 2.2. Measures

Sociodemographic data and self-report questionnaire were used.

*Job Demands and Job Resources* were measured using the Job Content Questionnaire (JCQ) [73]. It is one of the most widely used tools in assessing job demands and resources. It consists of three main scales (psychological demands, job decision latitude, and social support). Items are scored on a four-point Likert-scale, ranging from (1) “never” to (4) “always”. High scores indicate elevated appraisal of job demands and resources. The Psychological Demands (Cronbach’s α = 0.645) or workload subscale has nine items (e.g., “If I am interrupted, I have to finish the tasks later”). The Job Decision Latitude subscale (Cronbach’s α = 0.630) assesses the opportunities for workers to develop and implement their skills at work as well as their level of autonomy or degree of control over the tasks they perform. It consists of 9 items (e.g., “In my job, I am free to decide how to do the tasks”). Social support is measured through two subscales: The Social Support from coworkers subscale (Cronbach’s alpha = 0.753) that consists of six items (e.g., “I get along with my colleagues”) and the Social Support from supervisor scale (Cronbach’s alpha = 0.673) that includes five items (e.g., “The supervisor is concerned about our well-being”). High scores denote high appraisal of support.

*Exhaustion* (Cronbach’s α = 0.793) was measured using the 5-item subscale of Maslach Burnout Inventory-General Survey (MBI-GS) [74]. Items are answered on a seven-point Likert scale, ranging from 0 “never” to 6 “always”. High scores denote higher levels of Exhaustion (e.g., “I feel tired when I have to face another day at work”).

*Work Engagement* (Cronbach’s α = 0.893) was assessed using 6 items of Utrecht Work Engagement Scale Shortened Version (UWES) [65] corresponding to the core dimensions of this construct: vigor (three items, e.g., “I feel bursting with energy at work”), and dedication (three items, e.g., “I am proud of my job”). All items were rated on a seven-point scale ranging from 0 (“never”) to 6 (“always”). High scores indicate higher levels of work engagement.

*Job Satisfaction* (Cronbach’s α = 0.796) was measured using a survey developed by the first author of the present study [75] based on different studies with the intellectually disabled population [40,41]. This questionnaire consists of five indicators of job satisfaction [76]; for example, with the work system, or with salary. Items are answered on a 4-point Likert type scale, with (1) meaning “never”, and (4) meaning “always”. The higher the score, the higher the job satisfaction.

Several changes were introduced to enhance the understandability of the measures. First, the font size of the measures was enlarged to 14 points. Second, negative statements were rephrased into positive equivalents, and easy language and no ambiguous or complex phrasings were used, as the literature suggests [77,78]. Third, the administration procedure was modified, and measures were applied as an interview. Questions were read out loud to each respondent and presented by means of a set of cards. Fourth, as previous studies recommend [33,35,39,79], a separate set of cards with facial expressions (from a large frown through a broad grin), together with a color code (from green through orange to red) and corresponding labels were utilized to help utilize Likert-type responses.

### 2.3. Data Analysis

All the statistical analyses were performed using the software IBM SPSS 21 and AMOS 21. Assumptions were checked to ensure the adequacy of the analyses: high sample size, multivariate normality, linearity, and correlation between variables [80]. Confirmatory factor analysis (CFA) and goodness of fit indices were utilized. For confirmation of the adequacy of the model, we used the absolute fit indices; the chi-square statistic (X2) [81]; and the comparative fit index (CFI), with a reference value of 0.90 [82]. For confirmation within parsimony adjustment indices, we used the error of the root mean square approximation (RMSEA): the smaller its value, the better the fit, the reference value being 0.05 [83]. The Hoelter Index was also included to determine the adequacy of sample size. Finally, an analysis of invariance was carried out across groups. This is a hierarchical procedure that begins with an unconstrained level and continues by adding constraints successively. The logic of this procedure is to test the factorial homogeneity structure across groups, from a stage where all parameters do not need to be equal to a stage where they must be equal in a more restrictive way.

## 3. Results

### 3.1. Descriptive Statistics and Correlations

First, descriptive analyses were carried out, as depicted in Table 1. Skewness and kurtosis were <0.90 and >−0.90. Sex differences were examined across these variables. Differences did not reach the statistical level of significance, except for job decision latitude, where men (mean = 2.36; SD = 0.488) obtained lower scores than women (mean = 2.45; SD = 0.513): t(551) = 2.02; *p* < 0.05; d’ = 0.17. Moreover, Pearson’s correlation was carried out across the variables included.

Table 1 summarizes the main results. Note the scores for job demands and job resources are high. In contrast, exhaustion average is low. Work engagement is high, as is job satisfaction. With regard to correlations, psychological demands positively correlated with exhaustion and negatively correlated with job satisfaction, but not with work engagement. On the other hand, social support from colleagues and supervisors significantly correlated to each other and negatively correlated with exhaustion, while no significant association was found for decision latitude. Likewise, all resources were significantly and positively correlated with work engagement and job satisfaction.

### 3.2. Test of the JD-R Model

First, differences across inherent variables to the JD-R model were addressed across groups. A Student’s *t*-test for independent samples was carried out across sheltered workshop (*n* = 362) and supported employment *(n* = 192) groups (see Table 2). Differences for exhaustion and social support from supervisors were not statistically significant. Significantly higher scores were found in the remaining variables for the supported employment group.

Next, a linear multiple regression was carried out with job satisfaction as the dependent variable and the target variables under study as predictors. The group variable was included in the analysis as a dummy variable (1 = SW; 2 = SE). The adjusted R^2^ for the whole dataset was 0.47, and the resulting model was significant; F(7;.553) = 69.77; MSE = 23.63; *p* < 0.001. Table 3 depicts the coefficients and variables included in the model. It can be seen that work engagement was the strongest predictor of job satisfaction. In addition, low exhaustion, working in supported employment, having high social support from supervisor, and experiencing low psychological demands helped predict job satisfaction.

### 3.3. A Multi-Group Invariance Study on Intellectual Disability

Previous analysis suggested that some differences might occur in some of the variables under study. Since it could be of interest to address H3 and particularly H4, a structural equation modeling (SEM) approach was carried out, and invariance across groups was conducted. All variables under study were considered for this model. Even though job decision latitude and social support from coworkers did not predict job satisfaction, we considered the association among job resource variables to be more complex. For this reason, this variable was considered a second order, as supported by the literature reported in previous sections.

**Figure 1:**
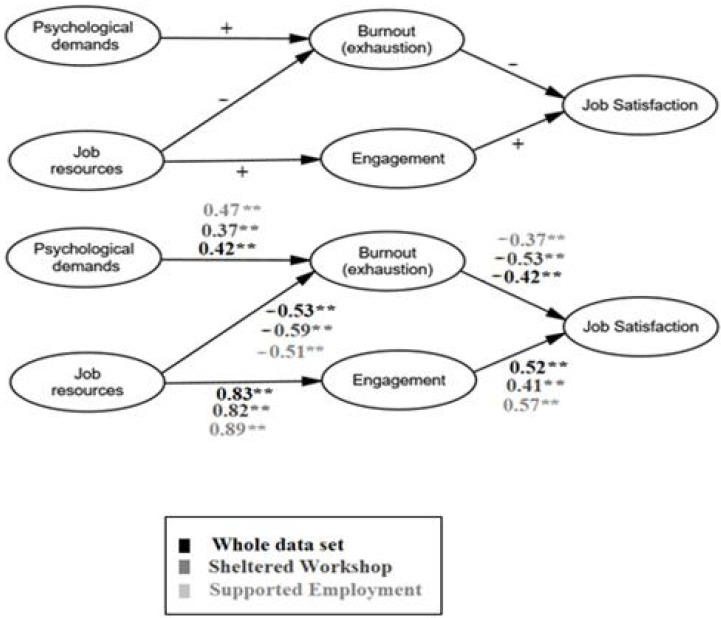
**Above**: the theoretical model and relationships. **Below**: the empirical model, the whole dataset in black, and shades of gray for the groups under study. Note: ** *p* < 0.001

Figure 1 depicts the main theoretical model. The goodness of fit was adequate (χ2 = 807.48; *p* < 0.001; χ2/df = 3.30; CFI = 0.894; RMSEA = 0.06; Hoelter 193–205). The SEM also supported H1 and H2 respectively, as well as previous analysis, as: (i) job demands were negatively related to job satisfaction through their impact on exhaustion via a health impairment process; (ii) and inverse relation was found for job resources, which were positively related to job satisfaction, through their impact on work engagement. Even if psychological demands increase burnout, job resources seem to mitigate it, as expected in H3.

Lastly, a multi-group analysis was carried out to examine the invariance in the model. As depicted in Figure 1, the model behaves similarly for both groups, as expected in H4.

When restrictions were added on factorial weights, metric invariance was achieved for both groups, but scalar invariance was not, as the Δ χ2 was much higher (see Table 4). This supports that the two-way process proposed in the JD-R model are similar for the two employment modalities up to a metric invariance, or factor weights for two groups. However, some items might be scored higher or lower in one of the groups, as commonly happens [84].

## 4. Discussion

The main objective of this study was to test the adequacy of the Job Demands–Resources (JD-R) model [57,58] in workers with intellectual disability belonging to the two most common employment options, namely, sheltered workshops and supported employment. The results allowed us to corroborate two of the main propositions of the JD-R model. Specifically, we found that in work environments, there are job demands and resources that impact the well-being and job satisfaction through burnout and engagement, respectively. These results provide support for the two-way process formulated in the JD-R model regarding the existence of two independent, although related, processes (health impairment and motivational) that explain the well-being of workers with intellectual disability. In fact, the model jointly explains 47% of the variance in job satisfaction. These results are consistent with those recently obtained by Ybema et al. [55]. Our study provides further evidence on the suitability of the JD-R theory in the prediction of job satisfaction using the structural equation procedure in a large sample of workers with ID. As far as we know, there are no previous studies that have used a multi-sample of workers with intellectual disability to contrast the core assumptions of the JD-R theory, this being one of the main contributions of this study.

In line with the health impairment process [58,61], and according to our first hypothesis, the results show that situations with high psychological demands, such as work overload, cause exhaustion for workers with ID that can lead to poorer job satisfaction. Previous studies have reached similar conclusions, demonstrating that prolonged exposure to job demands and the resulting burnout lead to poorer personal and organizational outcomes [40,55].

Regarding the motivational process [58,61], the results support the second hypothesis, confirming the two-way process. Furthermore, we found that together with work engagement, job resources are better predictors of job satisfaction than job demands. These results agree with previous studies [39]. They also stress the role of job resources in the prediction of job satisfaction and motivation, as postulated in the JD-R theory [57,58]. Our findings allow us to confirm that job resources and especially, work engagement, are key in the motivation of workers with ID, and they contribute to their well-being in the workplace.

Resources can also be helpful in mitigating burnout and exhaustion experienced by workers with intellectual disability, as stated in the third hypothesis. The existence of an inverse and significant association between job resources and exhaustion supported our predictions. However, contrary to our expectations, no association was found between job decision latitude and exhaustion. This result offers partial support to our hypothesis and is in line with some of the conclusions drawn by the authors of the JD-R theory about the weak negative impact that some resources have on burnout [61,62]. A possible explanation could be that job decision latitude was not perceived by ID workers as a job resource but rather as a job demand if they feel that they lack self-management skills. Excessive support provided by supervisors could also help explain these limitations [33]. In addition, some recent research did not find any significant association between job resources and burnout [55]. All of the above suggests the need for further research in this regard.

This study has demonstrated that the two processes established in the JD-R theory are valid and similar for the two employment modalities. Specifically, the results showed that job satisfaction is the result of the appraisal of the adequate balance between job demands and resources. The invariance model was reached both at the configural and metric level. However, in this case, as in many other multi-group studies, the invariance did not reach the scalar level [62,84]. This suggests that the way of responding and rating the items may be higher or lower depending on the group, thus reflecting differences between the individual responses to items. Similar results were also obtained in most studies that have contrasted the JD-R model in different groups of workers without disabilities [56,62,66].

Regarding the job satisfaction experienced by these employees, the results revealed the existence of significant differences in the two groups analyzed, with workers from sheltered workshops having lower job satisfaction. They also perceived higher psychological demands, fewer job (job decision latitude) and interpersonal (social support from colleagues) resources, and less work engagement. These results offer support to those in favor of transforming the initiatives of sheltered workshops into more inclusive employment services [85]. However, and in line with previous studies [38,39,40,41,42,53,54,86], most of the workers experienced high levels of job satisfaction. Although other studies did not find differences between work alternative and job satisfaction of employees with disabilities [38,39], the tasks that ID employees usually perform in the different work modalities could help explain the current findings. It could also be related to the concept of informed choice. People with disabilities must be provided with opportunities to exercise informed choice in decision-making and regarding employment services, to promote inclusion and integration into society [87]. In this regard, supported employment initiatives seem to offer more opportunities for choosing and feeling part of the community [88]. Furthermore, additional personal, contextual, or organizational factors (e.g., sociodemographic variables, personality traits, perceived self-efficacy, length of time in the position, job choice, salary, type of contract, etc.) could also be relevant to predict job satisfaction. Further studies on the different variables that could explain these differences are advisable.

It is also necessary to mention several limitations present in our study. First, participants were recruited using a non-probabilistic convenience sampling process, which limits the generalizability of our findings. In addition, levels of intellectual disability ranged from mild to moderate, so further studies are required to replicate current findings with the most severe levels of intellectual disability. A second limitation relates to its cross-sectional design. This implies that the observed associations in the model must be interpreted with caution and not establish causal inferences, since the existence of reciprocal relationships or inverse causalities could also be possible. Although longitudinal designs are desirable, the hypotheses tested in the present study require large datasets that are often difficult to collect in those research designs [70]. Utilizing shorter time intervals (for example, 2 to 6 months) could be a possible solution, although no longitudinal study has yet been carried out with this population that allows us to determine the best time interval.

Another limitation is the type of measures used. In our case, we have only used self-report measures administered by interview. Additional objective and psychophysiological measures could useful to corroborate the two processes established in the model. Thus, in the case of the pathway of deterioration in health, it could be interesting to include some psychophysiological record (e.g., blood pressure) to help identify the physiological responses to chronic stress situations. Regarding the motivational pathway, objective indicators (e.g., absenteeism, salary) could be considered. So far, studies that include this type of measure are scarce and nonexistent in the disability field. Daily registers could also be used to collect information on demands and resources, thus allowing analyzing their temporal variation and its effect on well-being and satisfaction, as some incipient studies have shown [55]. Likewise, studies using qualitative methodology could be useful to explore in depth the vision of workers with ID regarding other factors (e.g., choice of current job, experience in other jobs, view on the promotion of protected alternatives to more integrated alternatives, etc.) that could also be determinants of their job satisfaction.

Finally, the model explained 47% of the variance of job satisfaction. Therefore, more research is needed to determine other factors that may be involved in the job satisfaction of workers with intellectual disability. The inclusion of personality variables (e.g., self-efficacy, self-esteem, and optimism) as well as other job demands and resources not included in the model (e.g., role conflict, performance training, self-determination), together with other features such as gender, age, level of disability, additional coexisting disabilities, type and intensity of required supports, choice of employment, previous experience or length of time in the job, could help increase the explanatory power on the predictors of job satisfaction. The existence of psychometrically validated measures such as the Support Intensity Scale in the Spanish context may promote the inclusion of this variable in further studies [89].

Despite these limitations, the current findings have several practical implications both for improving the health and job satisfaction of employees with ID, and for organizations and professionals interested in promoting social inclusion and the quality of work life of these workers. First, different actions aimed at reducing the health impairment process in the presence of high job demands and low resources are suggested. One would be to seek an adequate match between the worker and job position. This means that, in addition to assessing the worker’s skills and abilities, workplace assessments must be carried out to determine the existing job demands [90]. Teaching strategies for preventing and coping with psychosocial risks and stress in the workplace would be another one of the actions aimed at improving the occupational health of these workers. Psychoeducation, relaxation techniques, problem-solving skills, time management, and dealing with dysfunctional thoughts could be effective strategies to reduce the impact of job demands on health and well-being.

Second, this study confirms the role that social support and work engagement play as elements that favor workplace well-being. Here, motivating workers through the recognition, acquisition, and development of job resources, both interpersonal (e.g., support from supervisors and colleagues) and personal or organizational (e.g., autonomy, variety of tasks, participation in decision-making) is of paramount importance to increase their satisfaction. The provision of adequate supports in the workplace will not only improve the functioning of the workers with ID, but it will also promote their personal and work outcomes as well as their quality of life [91]. In this study, social support from supervisors is the labor resource that contributes the most to explaining job satisfaction. There is no doubt about the importance of this type of support for any worker [56,57]. However, the way in which such support is provided needs to be underscored [38]. A too directive style and oriented toward the goals, or alternatively, being too overprotective will not allow workers with ID to explore or use their personal and work resources, which can also generate stress. For its part, adopting a participative leadership style will allow them to discover and activate other relevant personal and work resources (e.g., control over the task). Here, encouraging the participation of workers, offering them regular feedback on their performance, and listening to their opinions will result in greater self-efficacy and performance.

Another significant support comes from co-workers. In our study, it contributed to mitigate stress and is perceived differently depending on the job alternative. In supported employment, it is widely demonstrated that the more natural the supports, the better the benefits for the worker and for the organization [23]. In sheltered workshops, peer support is also valued as a key element to improve the quality of work life [75]. This type of support not only fulfills an instrumental function but also an emotional function, as it is useful in alleviating the impact of demands on stress and burnout [92]. Therefore, promoting social relations in the workplace, encouraging cooperative work, and enhancing the participation of workers in organized activities both inside and outside the work context are some initiatives that could favor personal relationships with coworkers.

Finally, work engagement constitutes a key element for achieving personal outcomes and work well-being. Therefore, providing stimulating and motivating work environments is crucial to improve job satisfaction. Fostering the personal resources of employees is an effective way to achieve this [93,94]. Allowing the worker to take the initiative and make changes in executing tasks, stimulating self-efficacy by reinforcing the achievements and skills of the person, and promoting optimism, hope, and resilience are some strategies to promote it. Likewise, enhancing self-determination by helping persons with disabilities to see their life and work as something that they can influence and act on would undoubtedly contribute to increase their motivation, which in turn will result in greater personal and organizational outcomes.

## 5. Conclusions

In the present study, we have demonstrated that Job Demands–Resources (JD-R) theory is a useful framework to explain the health and well-being of workers with intellectual disability. The study allowed us to demonstrate that certain job demands can deteriorate occupational health and lead to lower job satisfaction. Likewise, we have also demonstrated that certain job resources influence the motivation of the workers, increasing their enthusiasm and commitment to work, which has a positive impact on work well-being. The study also highlights the robustness of the model in two common employment alternatives in the field of intellectual disability.

## Figures and Tables

**Table 1:** Descriptive analysis on the variables of interest and Pearson’s correlation (*n* = 554).

	Mean (SD)	1	2	3	4	5	6	7
1. Psychological Demands	2.46 (0.50)	—						
2. Social Support Supervisor	2.96 (0.60)	0.076	—					
3. Social Support Coworkers	2.96 (0.59)	−0.094 *	0.389 **	—				
4. Job Decision Latitude	2.39 (0.50)	0.199 **	0.356 **	0.315 **	—			
5. Exhaustion	2.03 (1.51)	0.412 **	−0.220 **	−0.196 **	−0.048	—		
6. Work Engagement	4.03 (1.49)	−0.050	0.392 **	0.426 **	0.355 **	−0.425 **		
7. Job Satisfaction	2.89 (0.79)	−0.217 **	0.382 **	0.380 **	0.233 **	−0.463 **	0.533 **	—

Note. * *p* < 0.01; ** p < 0.001.

**Table 2:** Descriptive statistics across samples and Student’s *t*-test.

	Group	*n*	Mean	SD	*p*	Cohen’s d
Psychological Demands	SW	362	2.520	0.450	<0.001	0.33
	SE	192	2.354	0.589		
Social Support from Supervisor	SW	362	2.969	0.59	0.682	--
	SE	192	2.947	0.64		
Social Support from Coworkers	SW	362	2.845	0.591	<0.001	−0.58
	SE	192	3.175	0.520		
Job Decision Latitude	SW	362	2.309	0.451	<0.001	−0.51
	SE	192	2.557	0.546		
Exhaustion	SW	362	2.081	1.491	0.240	--
	SE	192	1.922	1.567		
Work Engagement	SW	362	3.734	1.519	<0.001	−0.59
	SE	192	4.593	1.275		
Job Satisfaction	SW	362	2.700	0.797	<0.001	−0.71
	SE	192	3.241	0.665		

Note. SW = Sheltered workshop; SE = Supported Employment.

**Table 3:** Variables included in the linear regression model in the prediction of job satisfaction.

Model	B	SE	β	*t*	*p*
Constant	1.340	0.207		6.475	<0.001
Psychological Demands	−0.129	0.057	−0.082	−2.251	0.025
Social Support Supervisor	0.252	0.049	0.193	5.193	<0.001
Social Support Coworkers	0.087	0.050	0.065	1.747	0.081
Job Decision Latitude	−0.022	0.058	−0.014	−0.381	0.703
Exhaustion	−0.118	0.020	−0.226	−5.814	<0.001
Work Engagement	0.175	0.022	0.328	8.100	<0.001
Group	0.332	0.058	0.199	5.720	<0.001

Note. SE = Standard error; B = Unstandardized; β = Standardized.

**Table 4:** Goodness-of-fit; multi-group analysis for Sheltered Workshop (*n* = 362) and Supported Employment samples (*n* = 192).

Model	χ^2^	d.f.	χ^2^/df	CFI	RMSEA	Δ χ^2^	Δdf	Decision
Model 1	1346.17	488	2.76	0.84	0.056	-	-	-
Model 2	1386.93	506	2.74	0.84	0.056	64.28	18	Accept
Model 3	1573.61	530	296	0.81	0.060	216.84 **	24	Reject

Note. CFI = Comparative Fit Index; RMSEA = Root Mean Square Error of Approximation; Model 1 = Configural Invariance; Model 2 = Full Metric Invariance; Model 3 = Full Metric and Scalar; ** *p* < 0.001.

## Data Availability

The data presented in this study are available on request from the corresponding author.
